# Decomposing the rural–urban differences in depression among multimorbid older patients in India: evidence from a cross-sectional study

**DOI:** 10.1186/s12888-023-05480-7

**Published:** 2024-01-22

**Authors:** Amiya Saha, Bittu Mandal, T. Muhammad, Waad Ali

**Affiliations:** 1 Department of Family & Generations, International Institute for Population Sciences, Mumbai, 400088 India; 2 School of Humanities and Social Sciences, Indian Institute of Technology, Indore, 453552 India; 3 Center for Healthy Aging, The Pennsylvania State University, University park, 16802 USA; 4 Department of Geography, Sultan Qaboos University, Muscat, 123 Oman

**Keywords:** Multimorbidity, Depression, Older adults, Rural–urban, LASI

## Abstract

**Background:**

In India, the prevalence of depression among older adults dealing with multiple health conditions varies between rural and urban areas due to disparities in healthcare access and cultural factors. The distinct patterns observed underscore the necessity for tailored research and interventions to address mental health inequalities among multimorbid older patients in diverse geographic contexts.

**Methods:**

This study used data from the Longitudinal Ageing Study in India (LASI) wave 1 (2017–18). A total of 7,608 adults aged ≥ 60 years who were diagnosed with two or more chronic conditions (such as hypertension, diabetes, cancer, chronic lung disease, chronic heart diseases, stroke, bone/joint disease, any neurological or psychiatric diseases, and high cholesterol) were included in this study. Descriptive statistics, bivariate analysis, logistic regression estimates, and Fairlie decomposition method were used to accomplish the study’s objectives.

**Results:**

The prevalence of depression among older adults with multimorbidity was 9.48% higher in rural areas (38.33%) than in urban areas (28.85%).. Older adults with multimorbidity belonging to the scheduled caste group were 40% more likely to experience depression. Moreover, those with multimorbidity and any form of disability in activities of daily living (ADL) were 93% more likely to experience depression than those without disability, whereas those with multimorbidity and perceived good general health were 65% less likely to suffer from depression than those with poor self-perceived health. Additionally, decomposition analysis revealed that education (35.99%), caste status (10.30%), IADL disability (19.30%), and perceived discrimination (24.25%) were the primary factors contributing to the differences in depression prevalence among older adults with multimorbidity between rural and urban areas.

**Conclusions:**

We found significant rural–urban differences in depression among older Indians with multimorbidity. The findings underscore the need for targeted interventions that address the unique challenges faced by older patients in rural areas, including lack of social capital, discrimination, and limited resources that enable access to healthcare services. Policymakers and healthcare professionals must collaboratively design and implement effective strategies to improve the mental health and overall well-being of rural older adults, particularly those with multiple comorbidities.

## Background

The growing issue of age-related multimorbidity and depression [1] in low- and middle-income countries (LMICs) poses a serious challenge to global health care systems [2]. Multimorbidity, which affects the majority of older adults, is the occurrence of two or more chronic diseases in an individual at the same time [3]. Depression [4], which is underdiagnosed and untreated along with other chronic diseases, has a negative impact on the overall health of individuals [5].

Findings from the burgeoning literature suggest a relationship between multimorbidity and depression, despite the lack of specific mechanisms between them [6]. Since they have a lower quality of life, more frequent use of medical services, and higher degrees of disability, pain, and cognitive impairment, people with multimorbidity are more susceptible to depressive disorders [6]. Barnett et al. (2012) suggested that depression with comorbidities covaries in a dose-dependent manner, such that an increase in the number of comorbid conditions is associated with a greater likelihood of depressive disorders and other mental health issues [3]. In addition, there is probably a statistically negative association between the prevalence of physical ailments and depression, and it was also evident from the study of Thombs et al. (2006) that subsequent depression is commonly associated with medical history [7].

According to recent estimates, 3.3 percent of Indians reported having depressive disorders, while nearly one in four had multimorbidity [8, 9]. Considering the situation in India, depression is a growing problem for individuals, especially older ones and is a substantial contributor to the future burden of the disease. The Global Burden of Disease Survey showed that those with multiple diseases were more likely to experience depressive symptoms than those without any diseases [10]. A recent study in India also reported that older adults with more chronic conditions are more vulnerable to depressive symptoms [11]. A study by Shubham et al. (2023) [12] investigated urban–rural differences in depressive symptoms among older adults with an urban disadvantage in India. Previous studies have also shown that individuals residing in urban areas may be more likely to experience depression than those living in rural areas because of the diminution in social connections and social isolation [13, 14]. The most recent studies on multimorbidity and associated depression among older adults in India were conducted in community settings [15–18].

However, researchers have been intrigued by the potential factors contributing to the differences in mental disorders between rural and urban areas [19–24]. A cross-sectional study that pooled data from a multicentre randomized controlled clinical trial in Canada found that adults with multiple chronic conditions and access to healthcare services in rural areas exhibited better mental health than their urban counterparts [24]. The difference in services between rural and urban areas is often of concern, making studies examining the differences between rural and urban depression important [25]. Moreover, a previous study found that urban–rural differences in depression [12] vary by country; therefore, it is suggested to conduct country-specific studies on urban–rural differences in depression to predict the essential approaches to managing the conditions.

Although there is a substantial amount of research on the risk factors and urban–rural gradients in depression among older population, none of these studies have examined the differences between urban and rural depression in older people with multiple chronic conditions. Thus, it is imperative to examine the differences between urban and rural areas regarding the mental health needs of aging individuals with multiple chronic conditions to effectively plan and implement mental health services and programs for rural communities where such needs may be insufficiently met. This study investigated the rural–urban gradient of depression among older Indian adults. The factors that contribute to rural–urban differences in the prevalence of depression among older adults with multiple chronic conditions were also decomposed in this study.

## Materials and methods

### Data source

This study utilized data from the Longitudinal Ageing Study in India (LASI) wave 1, a large-scale survey specifically for older adults aged 45 years and above, which is a longitudinal study of health and ageing [26]. Eventually, a unit of observation of LASI will be LASI’s age-eligible households (LEH) [26]. The LASI from the chosen families consisted of all males and females who were 45 years of age or older, as well as their spouses, irrespective of their age [26]. LASI offers reliable, organized, and ongoing scientific data on the population of older persons (aged 45 and over), including their physical, social, psychological, and economic well-being [26]. The targeted sample was chosen using a multistage stratified area probability cluster sampling design and consisted of non-institutionalized inhabitants of India's 31 states and six Union Territories [26].

The total household response rate was 96%, while the overall individual response rate was 87% (rural,89.6%; urban,83.6%) [26]. Each household and age-eligible individual provided a written informed consent. Four consent forms—household informed consent, individual informed consent, consent for the collection of blood samples for storage and subsequent use (DBS), and proxy consent—were used in compliance with the protection of human subjects [26]. The Indian Council of Medical Research (ICMR) provided the required instructions and ethical clearance to conduct the LASI.

### Selection of the study sample

**Fig. 1:**
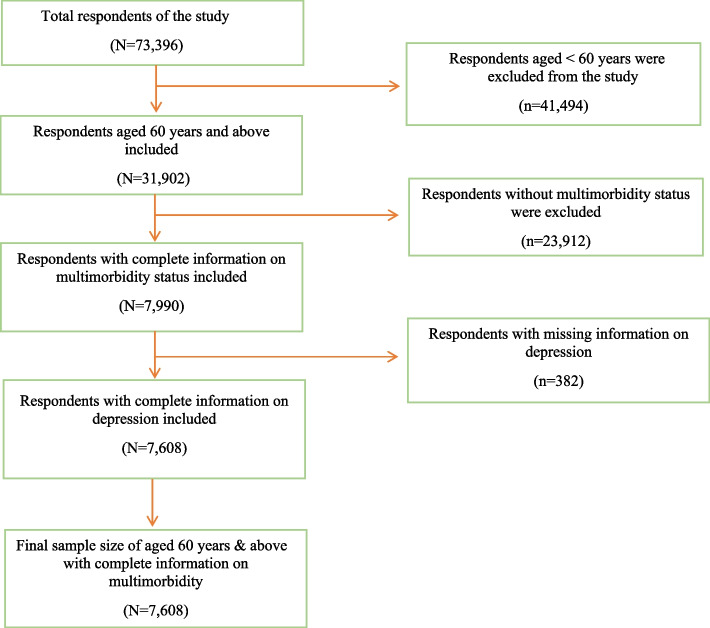
Selection criteria of the sample study

In this study, we included adults aged 60 years and above with comprehensive information on reported depression. Figure 1 shows detailed information on the inclusion and exclusion criteria for the study sample of older adults with multimorbidities. The presence of two or more chronic diseases is referred to as multimorbidity [27]. Nine distinct chronic diseases were covered by the LASI survey: (1) hypertension or high blood pressure, (2) diabetes, (3) cancer, (4) any chronic lung disease such as asthma, (5) chronic heart diseases, (6) stroke, (7) bone/joint disease, (8) any neurological or psychiatric diseases, and (9) high cholesterol [26]. These self-reports of chronic diseases were diagnosed [28] as was assessed through the question, "Has any health professional ever diagnosed you with the following chronic conditions or diseases?" [29]. An individual was coded as "No multimorbidity" if they had only one chronic disease or none at all and "Multimorbidity" if they had two or more chronic diseases [29]. Finally, the sample in this study included 7,608 older adults aged 60 years and above living with multiple chronic conditions. Among the study sample, 56.23% older adults resided in rural areas (in villages with a size that varies from 0–10,000 population) and 43.77% older adults resided in urban areas (in towns, wards and Census Enumeration Blocks).

### Variable description

#### Outcome variable

The outcome of interest i.e., depressive symptoms was measured based on the Centre for Epidemiologic Studies Depression Scale (CES-D-10) among older adults, and it has four scale options, ranging from (1) rarely or never (< 1 day) to (4) most of the time (5–7 days). Respondents were asked ten different questions regarding their experiences over the past week, including difficulty concentrating, feeling depressed, low energy, afraid of something, alone, irritated by things, everything is an effort, and feeling cheerful, hopeful, and satisfied [26]. Among the 10 items on the scale, the first seven were based on negative symptoms, and the final three on positive symptoms. Those who responded to negative symptoms by stating "rarely or never (1 day)" and "occasionally (1 or 2 days)" were given a zero score, while the other two categories were coded as one. In addition, when positive symptoms were present, scoring was reversed. The composite score spanned a scale of 0 to 10, where a value of four or higher was considered indicative of depression [30].

The detailed measurements of the explanatory variables in this study are provided in Table 1.

**Table 1:** Description of the other factors included in the study, Longitudinal Aging Study (LASI) Wave 1, India 2017–18

Socio-demographic		
	**Categories**	**Description of the category**
**Age**	Young-old (60–69 years)	Age of the respondents were available in "young-old (60–69 years)," "old-old (70–79 years)," and "oldest-old (80 years & above)" [31]
	old-old (70–79 years)	
	oldest-old (80 years & above)	
**Sex**	Male	Sex of the respondent was available in male–female categories [26]
	Female	
**Place of residence**	Rural	Place of residence (rural/urban) was determined according to the administrative division of India followed in Census of India, 2011. Households in urban areas included those in towns, wards and Census Enumeration Blocks whereas, households in rural areas include those in villages (size varies from 0–10,000 population [32]
	Urban	
**Education**	No education/ primary not completed	There were four categories for educational status: No education/ primary not completed," "Primary," "Secondary," and "Higher" [33]
	Primary	
	Secondary	
	Higher	
**Religion**	Hindu	Religion was categorized into Hindu, Muslim, Christian, and others [34]
	Muslim	
	Christian	
	Others	
**Caste**	Scheduled castes (SC)	Caste was coded as Scheduled castes (SC), Scheduled Tribes (ST), Other Backward Class (OBC) and others. SC and ST are India's most economically and socially disadvantaged groups. According to the Hindu caste system, the ST contains a segment of the population that is socially isolated and has a low economic position. People who were "educationally, economically, and socially backward" are classified as OBC. In the old caste order, the OBC is seen as being at the bottom yet somewhat above the most disadvantaged communities. The “other” caste category is identified as those having higher social standings [35]
	Scheduled Tribes (ST)	
	Other backward classes (OBC)	
	Others	
***Health and behavioural factors***		
**ADL disability**	Yes	In the individual schedule, ADL consists of difficulties with six activities related to dressing which include *putting on chappals or shoes, walking across a room, difficulties in bathing, eating, getting in or out of bed, and using the toilet, including getting up and down*. Further, combining these six ADLs into one variable, we constructed a variable coded as "no ADL" if the respondent faced no difficulty in performing any ADLs and "yes" if respondents faced any difficulty in performing any ADL [15, 32, 36]
	No	
**IADL disability**	Yes	IADL consisted of seven difficulties related to instrumental activities such as *difficulty in preparing a hot meal (cooking and serving), shopping for groceries, making telephone calls, taking medications, doing work around the house or garden, managing money, such as paying bills and keeping track of expenses and getting around or finding address in an unfamiliar place*. IADLs were also recoded as "no IADL" if the respondent faced no difficulty in performing any IADLs and "yes" if respondents having any difficulty in performing any IADL [15, 32, 36]
	No	
**Self-rated health**	Very	Self-rated health was measured on the basis of one question, which was, *overall, how is your health in general*? which includes five categories for responses, i.e., "Very poor," "Poor," "Fair," "Good," and "Very good" [26, 32, 36, 37]
	Poor	
	Fair	
	Good	
	Very good	
**Pain**	Yes	In order to define pain, participants were asked, "*Are you often troubled with pain*?" then it was coded as "no" and "yes" [15]
	No	
**Body Mass Index**	Underweight	The study focused on assessing the body mass index (BMI), which is a measure of weight in relation to height, among elderly participants. The BMI values were determined using the height and weight measurements of the respondents. The BMI results were then classified according to the World Health Organization's classification system, which categorizes individuals as underweight (BMI < 18.5 kg/m2), normal weight (BMI 18.5–24.9 kg/m2), overweight (BMI 25.0–29.9 kg/m2), and obese (BMI ≥ 30.0 kg/m2) [38–40]
	Normal	
	Overweight	
	Obese	
	Missing	
**Physical activity status**	Never	Three distinct classifications of physical activity were established: frequent (daily), infrequent (more than once a week, once a week, or one to three times a month), and never. To evaluate physical activity, individuals were asked the following question: "How frequently do you engage in sports or vigorous activities, such as running, swimming, going to the gym, cycling, or performing physically demanding tasks like digging or lifting heavy objects, chopping wood, or engaging in farm work, fast bicycling, or cycling with heavy loads?" [26, 31]
	Frequent	
	Rare	
**Tobacco consumption**	Yes	Tobacco usage was assessed through survey questions inquiring about past experiences with smoking tobacco products (e.g., cigarettes, bidis, cigars, hookahs, cheroots) and the use of smokeless tobacco (e.g., chewing tobacco, gutka, pan masala, etc.). Participant responses were coded as either "yes" or "no" [26]
	No	
**Alcohol consumption**	Yes	Similarly, alcohol consumption was evaluated by asking participants if they had ever consumed alcoholic beverages, including beer, wine, liquor, or country liquor. Responses were then coded as either "yes" or "no" [26]
	No	
**Health insurance coverage**	Yes	Health insurance coverage was coded as binary response “yes and “no” [26]
	No	
**Psycho-social factors**		
**Perceived discrimination**	Yes	The perception of discrimination was assessed using six questions, including statements such as, "You receive less courtesy or respect compared to others," "People treat you as if you are not intelligent," "People behave as if they are fearful of you," and "You experience threats or harassment." In addition, respondents' answers were classified into binary categories: "yes" if they reported experiencing discrimination on an almost daily basis, almost every day, a few times a month, or less than once a year, and "no" if they indicated never experiencing discrimination [26]
	No	
**Marital status**	Currently in union	Current research has classified marital status into binary classification, including currently in union and currently not in union [15, 32]. We do not prioritize examining the other non-married categories, despite recognizing that the association between different marital status categories may vary. Consequently, we have simplified the classification of marital status in our study by adopting a binary approach. Those who reported being "currently in union" to indicate their married status, while all other categories such as widowed, never married, separated, divorced, and deserted are consolidated as "currently not in union."
	Currently not in union	
**Working status**	Working	Furthermore, working status was recoded as "Working," "Retired," and "Not working" [31] in this study
	Retired	
	Not working	
**Community engagement**	Yes	Community engagement was assessed through the process of coding responses to survey questions regarding affiliation with social organizations, religious groups, clubs, or societies. The participants' responses were classified into two categories, namely "yes" and "no" [26]
	No	
**Household factors**		
**MPCE quintile**	Poorest	Using information on household consumption data, the monthly per capita consumption expenditure (MPCE) quintile has been assessed. The sample households were surveyed using sets of 11 and 29 questions on spending on food and non-food items, respectively. Food expenditures were collected during the seven-day reference period, whilst non-food expenditures were collected over 30-day and 365-day reference periods. Using 30-day reference period, expenses for both food and non-food items were standardised. The MPCE is calculated and used as a summary indicator of consumption. The variable was further divided into five quintiles, i.e., from poorest to richest [26]
	Poor	
	Middle	
	Richer	
	Richest	
**Regions**	North	The region was coded as "North," "West," "Northeast," "East," "Central" and "South" [26, 32] in this study
	West	
	Northeast	
	East	
	Central	
	South	

### Statistical analysis

Descriptive statistics and bivariate analysis have been used to compute the prevalence of depression with multimorbidity by socio-demographic, health-behavioural, psychosocial and household factors. The prevalence of depression among older adults with multimorbidity has been presented separately for rural and urban areas and conducted two sample proportion tests to compare the proportion of two independent groups of older patients in rural and urban areas [41]. Further, we performed binary logistic regression analysis [42] in the subsample of urban and rural to examine the difference in the magnitude of the associations between various background characteristics and depression among older adults with multimorbidity. All the results from the logistic regression analyses have been presented in the form of an odds ratio (OR) with 95% confidence interval (CI).

Following that, we used Fairlie decomposition to figure out the explanatory variables that contributed to the rural–urban difference in depression. The Blinder-Oaxaca decomposition is a frequently used method to identify and measure the variables associated with differences in the mean level of outcome between groups. While the Blinder-Oaxaca technique is used in linear models, the Fairlie, (2005) technique is suitable for non-linear models. The Fairlie decomposition method is a straightforward approach employed to estimate outcomes from a logit or probit model, originally introduced by Fairlie in 1999 [43]. The results of the decomposition have been explained in terms of coefficient and percent contribution by socio-demographic, health and behavioural, psychosocial and household factors. According to the Fairlie decomposition for a non-linear equation, Y = (^*kβ*^) can be written as,

$$\begin{array}{c}{{\varvec{y}}}^{-{\varvec{R}}}-{{\varvec{y}}}^{-{\varvec{U}}}=\left[\sum_{i=1}^{{N}^{R}} \frac{F\left({k}_{i}^{R}{\beta }^{-R}\right)}{{N}^{R}}-\sum_{i=1}^{{N}^{R}} \frac{F\left({k}_{i}^{U}{\beta }^{-R}\right)}{{N}^{U}}\right]+\left[\sum_{i=1}^{{N}^{v}} \frac{F\left({k}_{i}^{U}{\beta }^{-R}\right)}{{N}^{U}}-\sum_{i=1}^{{N}^{U}} \frac{F\left({k}_{i}^{U}{\beta }^{-U}\right)}{{N}^{U}}\right]\\ \\ \end{array}$$

where *N *^*R*^ and *N *^*U*^ indicate the sample size for rural and urban respectively, ${y}^{-R}$ and ${y}^{-U}$ are the average probability of the binary outcome of the interest (i.e., depression) for the group rural and urban, *F* is the cumulative distribution function from the logistic estimates, ${k}_{i}^{R}$ and ${k}_{i}^{U}$ are the set of the average value of the independent variable and ${\beta }^{-R}$ and ${\beta }^{-U}$ are the estimates of the beta coefficient for the rural and urban, respectively. All the analyses were conducted using STATA version 17.0 (Stata Corp, LP, college station, Texas) and sample weights were applied to adjust the effect of complex survey design.

## Results

### Background characteristics of the study population

Table 2 presents the demographic and socioeconomic characteristics of the respondents stratified by rural–urban sector in India. Among rural residents, little more than 56% aged 60–69 years and around 13% aged 80 + years whereas, in urban areas, 58% aged 60–69 years and 8.11% aged 80 + years. Notably, a significant percentage of both rural (70.58%) and urban (37.19%) residents lacked primary education, indicating high levels of illiteracy. When it came to obesity, urban dwellers (16.19%) had a higher prevalence compared to their rural counterparts (7.69%). In rural areas 35.69% and 62.71% were living with ADL and IADL difficulties, however, it was 25.95% and 48.79% in urban areas, respectively. Furthermore, 34.50% and 28.06% of the rural and urban respondents reported poor SRH, respectively. 50.11% and 40.78% of older adults residing in rural and urban areas were experiencing pain, respectively. 76.34% and 80.33% of the rural and urban older adults were not physically active, respectively. 13.05% rural and 10.58% urban older adults found be consuming alcohol. Additionally, 38.16%, and 27.12% of rural older adults were widowed, and never worked, respectively. Further, in urban areas 39.38% were widowed, and 37.10% were currently not working.

**Table 2:** Sample distribution by background characteristics among multimorbid older adults in rural and urban India (*n* = 7,608)

Background characteristics	Rural		Urban		*p value*
	**Sample**	**Percentage**	**Sample**	**Percentage**	
**Sociodemographic**					
**Age**					≤ 0.001
Young-old (60–69 years)	2,402	56.15	1,940	58.27	
Old-old (70–79 years)	1,320	30.86	1,120	33.62	
Oldest-old (80 + years)	556	12.99	270	8.11	
**Sex**					≤ 0.005
Male	1,961	45.85	1,373	41.24	
Female	2,317	54.15	1,957	58.76	
**Education**					≤ 0.001
No education/ Primary not completed	3,019	70.58	1,238	37.19	
Primary	525	12.27	523	15.7	
Secondary	261	6.1	388	11.66	
Higher	473	11.05	1,181	35.46	
**Religion**					≤ 0.001
Hindu	3,425	80.07	2,662	79.93	
Muslim	457	10.68	454	13.63	
Christian	176	4.1	111	3.34	
Others	220	5.15	103	3.1	
**Caste**					≤ 0.001
Scheduled caste (SC)	869	20.31	305	9.16	
Scheduled tribe (ST)	226	5.29	51	1.52	
Other backward class (OBC)	2,004	46.84	1,616	48.53	
Others	1,179	27.57	1,358	40.79	
**Health and behavioural factors**					
**ADL disability**					0.090
No	2,751	64.31	2,466	74.05	
Yes	1,527	35.69	864	25.95	
**IADL disability**					≤ 0.001
No	1,595	37.29	1,705	51.21	
Yes	2,683	62.71	1,625	48.79	
**Self-rated health**					≤ 0.001
Very poor	335	7.83	149	4.47	
Poor	1,476	34.5	934	28.06	
Fair	1,799	42.05	1,547	46.47	
Good	597	13.97	608	18.26	
Very Good	71	1.65	92	2.75	
**Pain**					≤ 0.001
No	2,134	49.89	1,972	59.22	
Yes	2,144	50.11	1,358	40.78	
**Body Mass Index**					≤ 0.001
Underweight	825	19.29	135	4.06	
Normal	1,913	44.72	1,271	38.18	
Overweight	862	20.16	1,053	31.62	
Obese	329	7.69	539	16.19	
Missing	348	8.14	332	9.96	
**Physical activity status**					≤ 0.001
Never	3,266	76.34	2,675	80.33	
Frequent	753	17.6	510	15.3	
Rare	259	6.06	145	4.37	
**Tobacco consumption**					≤ 0.001
No	2,566	59.99	2,577	77.38	
Yes	1,712	40.01	753	22.62	
**Alcohol consumption**					≤ 0.001
No	3,720	86.95	2,978	89.42	
Yes	558	13.05	352	10.58	
**Health insurance coverage**					
No	3,387	79.17	2,750	82.59	≤ 0.001
Yes	891	20.83	580	17.41	
**Psycho-social factors**					
**Perceived discrimination**					≤ 0.001
No	3,446	80.55	2,767	83.08	
Yes	832	19.45	563	16.92	
**Marital Status**					0.053
Currently in union	2,646	61.84	1,913	57.46	
Currently not in union	1,632	38.16	1,416	42.54	
**Working status**					≤ 0.001
Never worked	1,160	27.12	1,447	43.45	
Retired	2,209	51.64	1,421	42.67	
Currently working	909	21.25	462	13.89	
**Ill treatment**					≤ 0.001
No	4,009	93.72	3,203	96.19	
Yes	269	6.28	127	3.81	
**Community involvement**					0.270
No	4,045	94.55	3,164	95.0	
Yes	233	5.45	167	5.0	
**Household factors**					
**MPCE quintile**					≤ 0.001
Poorest	616	14.41	527	15.81	
Poor	834	19.51	574	17.23	
Middle	905	21.14	580	17.41	
Richer	879	20.55	811	24.36	
Richest	1,043	24.39	839	25.18	
**Region**					≤ 0.001
North	627	14.66	368	11.05	
West	709	16.58	803	24.12	
Northeast	100	2.33	54	1.61	
East	1,181	27.61	516	15.51	
Central	576	13.46	293	8.81	
South	1,085	25.36	1,296	38.9	
**Total**	**4,278**	56.23	**3,330**	43.77	

*LASI* Provided sampling weights were applied, *ADL* Activities of Daily Living, *IADL* Instrumental Activities of Daily Living, *MPCE* Monthly Per Capita Consumption Expenditure

### Rural–urban differences in the prevalence of depression among multimorbid older adults

Table 3 presents an analysis of depression prevalence among older adults with multimorbidity based on various background characteristics. Specifically, older adults aged 80 and above residing in rural areas (49.12%) reported a higher prevalence of depression compared to their urban counterparts (32.73%), indicating a substantial difference of 16.39% (*p* ≤ *0.005*). Furthermore, older adults with multimorbidity having secondary education exhibited a considerably greater disparity in depression prevalence between rural and urban areas, with rates of 37.05% and 16.64%, respectively. Older adults with multimorbidity experiencing ADL and IADL disabilities also reported a higher disparity in the prevalence of depression, with differences of 12.28% (*p* ≤ *0.001*) and 13.41% (*p* ≤ *0.001*), respectively. A total of 44.5% rural and 36.11% urban dwellers with multimorbidity reported pain. Moreover, among behavioural factors, significant higher urban–rural differences were found among older adults with multimorbidity who rarely engage in any physical activity, indicating a difference of 25.05% (*p* ≤ *0.001*). Significant rural–urban differences in depression prevalence were also observed among older adults with multimorbidity who were not in marital union (45.62% vs. 28.07%), those who were never worked (42.93% vs. 27.04%), individuals belonging to the poorest strata (48.79% vs. 33.90%), and those residing in the northeast region of India (24.62% vs. 10.31%).

**Table 3:** Rural urban differences in the prevalence of depression among multimorbid older adults by various factors in India, 2017–18

Factors	Rural (%)	Urban (%)	Difference	*p value*
**Sociodemographic**				
**Age**				
Young-old (60–69 years)	35.06	28.48	6.58	≤ 0.001
Old-old (70–79 years)	39.73	28.55	11.18	≤ 0.005
Oldest-old (80 + years)	49.12	32.73	16.39	≤ 0.005
**Sex**				
Male	35.35	28.50	6.85	≤ 0.001
Female	40.86	29.10	11.76	≤ 0.001
**Education**				
No education/ Primary not completed	42.42	35.28	7.14	≤ 0.001
Primary	29.17	32.71	3.54	0.498
Secondary	37.05	16.64	20.41	0.116
Higher	23.06	24.42	1.36	0.976
**Religion**				
Hindu	38.83	29.02	9.81	≤ 0.001
Muslim	41.37	30.4	10.97	≤ 0.001
Christian	32.64	25.24	7.40	≤ 0.005
Others	28.84	21.67	7.17	0.450
**Caste**				
Scheduled caste (SC)	49.63	33.31	16.32	≤ 0.005
Scheduled tribe (ST)	34.91	20.43	14.48	< 0.001
Other backward class (OBC)	35.63	27.27	8.36	≤ 0.005
Others	35.24	30.04	5.20	0.042
**Health and behavioural factors**				
**ADL disability**				
No	30.38	24.82	5.56	< 0.001
Yes	52.65	40.37	12.28	< 0.001
**IADL disability**				
No	26.51	25.9	0.61	0.045
Yes	45.36	31.95	13.41	< 0.001
**Self-rated health**				
Very poor	63.72	62.59	1.13	0.421
Poor	45.71	38.19	7.52	≤ 0.005
Fair	31.80	22.42	9.38	≤ 0.005
Good	27.18	24.18	3.00	≤ 0.005
Very Good	24.12	18.41	5.71	≤ 0.05
**Pain**				
No	32.13	23.85	8.28	≤ 0.001
Yes	44.5	36.11	8.39	≤ 0.001
**Body Mass Index**				
Underweight	53.56	41.65	11.91	≤ 0.005
Normal	36.9	29.34	7.56	≤ 0.005
Overweight	28.76	29.05	0.29	0.093
Obese	21.53	21.72	0.19	0.295
Missing	49.66	32.72	16.94	≤ 0.001
**Physical activity status**				
Never	39.82	27.41	12.41	≤ 0.001
Frequent	32.06	41.03	8.97	0.221
Rare	37.75	12.70	25.05	≤ 0.050
**Tobacco consumption**				
No	36.26	28.32	7.94	≤ 0.001
Yes	41.43	30.68	10.75	≤ 0.001
**Alcohol consumption**				
No	39.01	29.2	9.81	≤ 0.001
Yes	33.81	25.89	7.92	0.141
**Health insurance coverage**				
No	39.89	29.35	10.54	≤ 0.001
Yes	32.41	26.49	5.92	≤ 0.05
**Psycho-social factors**				
**Perceived discrimination**				
No	34.66	27.06	7.6	≤ 0.001
Yes	53.54	37.62	15.92	0.236
**Marital Status**				
Currently in union	33.83	29.43	4.4	≤ 0.001
Currently not in union	45.62	28.07	17.55	≤ 0.001
**Working status**				
Never worked	42.93	27.04	15.89	≤ 0.001
Retired	39.31	29.33	9.98	≤ 0.001
Currently working	30.07	33.03	2.96	0.521
**Ill treatment**				
No	36.93	27.82	9.11	≤ 0.001
Yes	59.23	54.9	4.33	0.962
**Community involvement**				
No	38.6	29.33	9.27	≤ 0.001
Yes	33.6	19.69	13.91	≤ 0.005
**Household factors**				
**MPCE quintile**				
Poorest	48.79	33.9	14.89	≤ 0.001
Poor	35.69	33.27	2.42	≤ 0.05
Middle	41.10	29.53	11.57	≤ 0.001
Richer	36.46	25.05	11.41	≤ 0.005
Richest	33.43	25.87	7.56	0.094
**Region**				
North	33.60	32.91	0.69	0.062
West	26.01	21.07	4.94	≤ 0.005
Northeast	24.62	10.31	14.31	≤ 0.05
East	46.26	37.89	8.37	0.512
Central	42.79	35.82	6.97	0.052
South	39.39	28.1	11.29	≤ 0.001

*LASI* Provided sampling weights were applied, *ADL* Activities of Daily Living, *IADL* Instrumental Activities of Daily Living, *MPCE* Monthly Per Capita Consumption Expenditure; *p* value based on two sample proportion tests

**Fig. 2:**
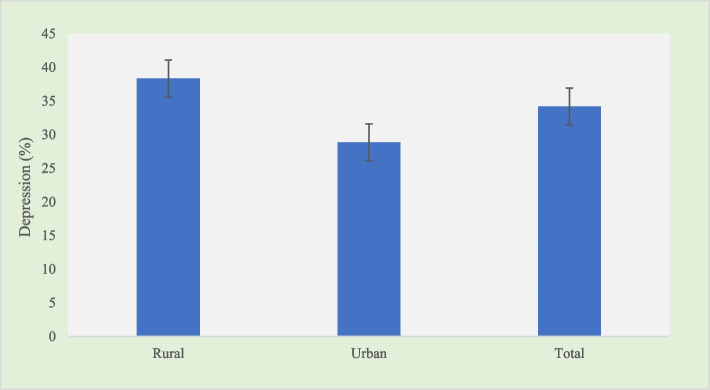
Prevalence of depression among multimorbid older adults in India, 2017–18

The greater prevalence of depression was observed among older adults with multimorbidity living in rural areas (38.33%) than urban areas (28.85%) (Fig. 2).

### Estimates of multivariable logistic regression

**Fig. 3:**
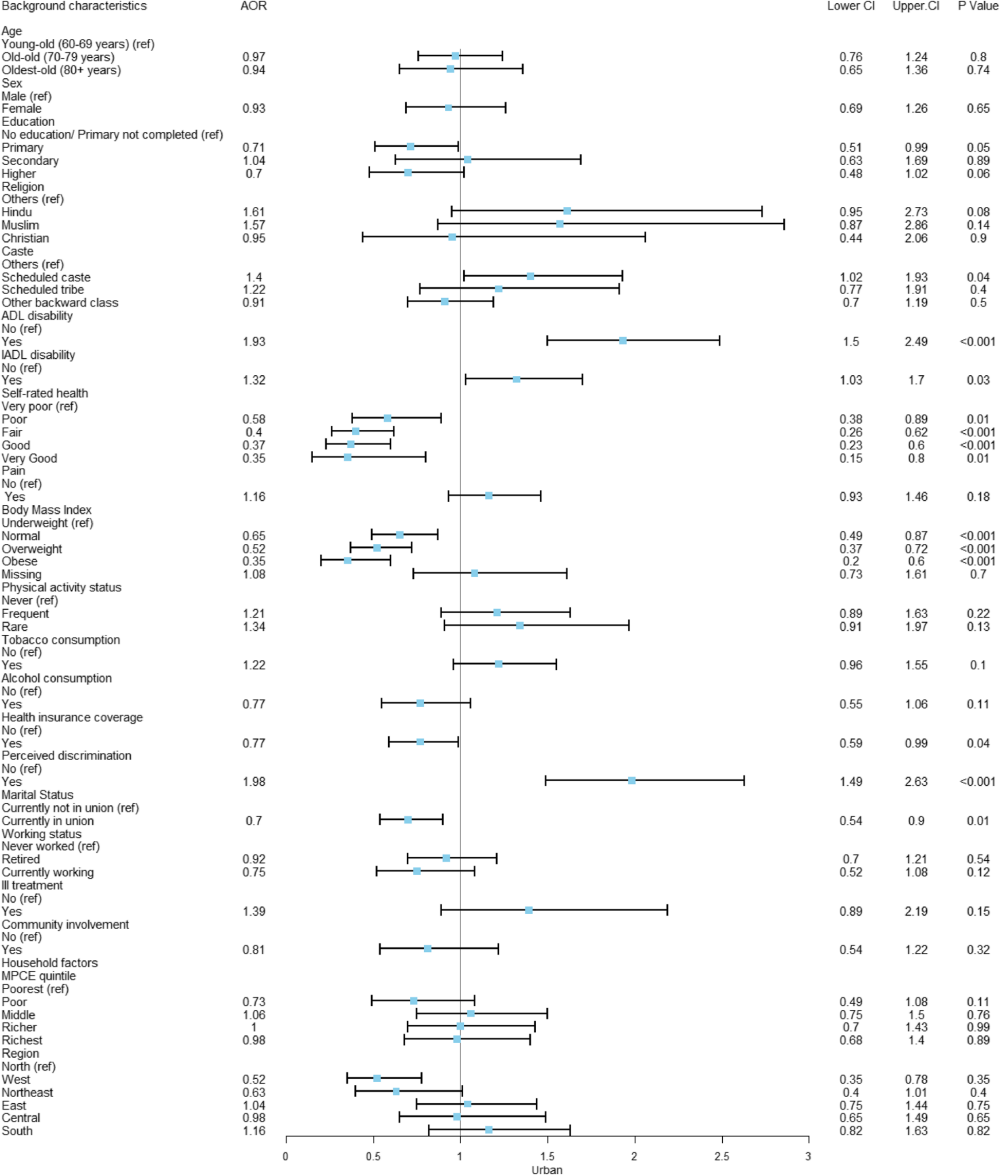
Adjusted odds ratio explaining the rural–urban differences of depression among multimorbid older adults in rural India (*n* = 7,608)

**Fig. 4:**
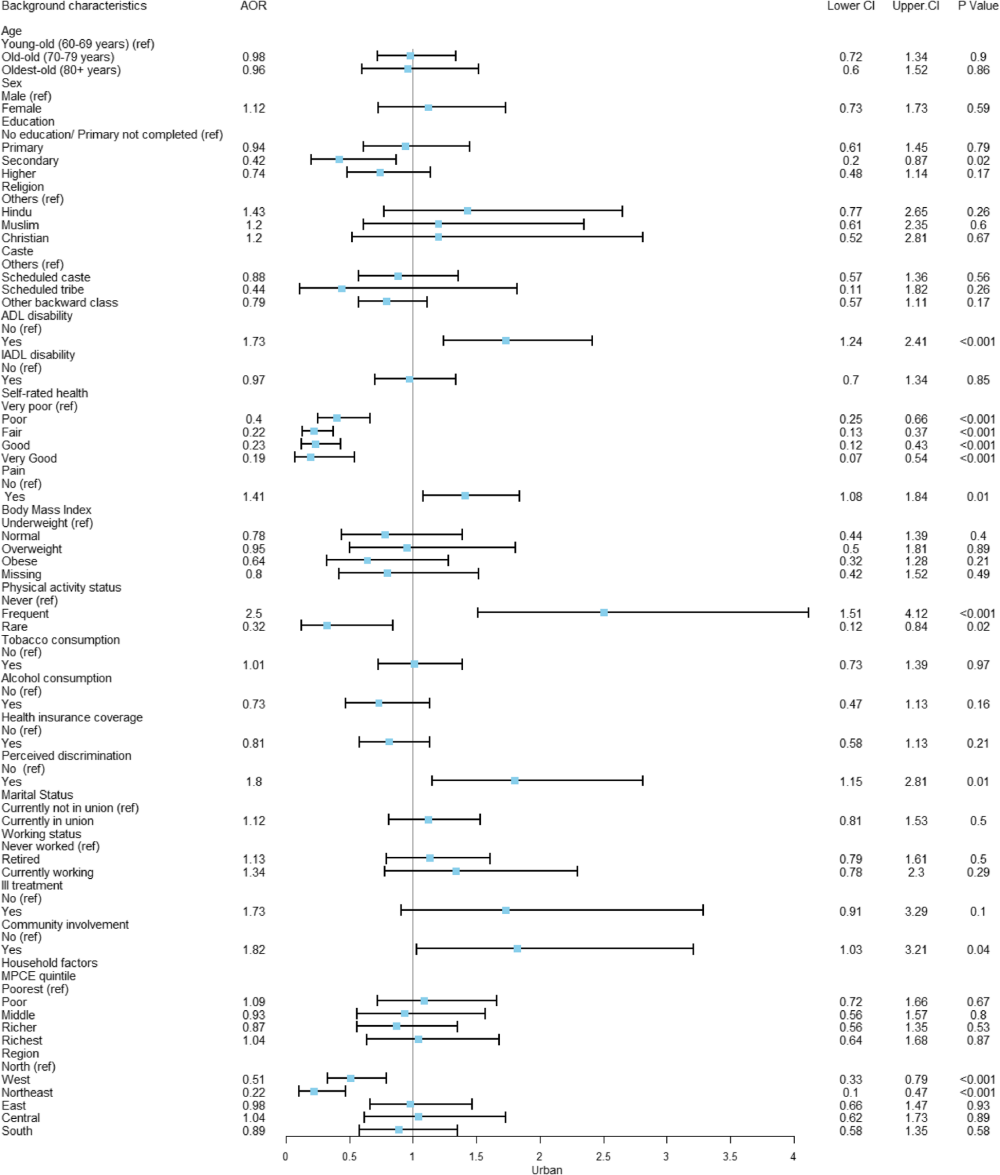
Adjusted odds ratio explaining the rural–urban differences of depression among multimorbid older adults in urban India (*n* = 7,608)

Figures 3 and 4 presents the risk factors of depression among older adults with multimorbidity, separately for rural and urban areas. The findings presented in Fig. 3 indicate that in rural areas, older adults with primary education are significantly less likely to experience depression compared to those who are illiterate (AOR: 0.71; CI: 0.51- 0.99). Older adults facing difficulties in ADL are 1.93 times more likely to suffer from depression (AOR: 1.93; CI: 1.50–2.49), while those with IADL difficulties are 1.32 times more likely to experience depression (AOR: 1.32; CI: 1.03–1.70). Additionally, older adults who rate their overall health as very good are 0.65 times less likely to be depressed compared to those who report very poor self-rated health (AOR: 0.35; CI: 0.15–0.80). In terms of weight, compared to underweight respondents, obese individuals (AOR: 0.35; CI: 0.20–0.60) and overweight individuals (AOR: 0.52; CI: 0.37–0.72) are less likely to experience depression. Furthermore, older adults who are in a current marital union are 0.30 times less likely to suffer from depression compared to their counterparts (AOR: 0.70; CI: 0.54–0.90).

In urban areas (Fig. 4), older adults with difficulties in ADL have a 1.73 times higher likelihood of experiencing depression (AOR: 1.73; CI: 1.24–2.41). Older adults who report very poor self-rated health are more likely to suffer from depression compared to those who report very good self-rated health (AOR: 0.19; CI: 0.07–0.54). Additionally, older adults who rarely engage in any form of physical activity are less likely to develop depression compared to those who never participate in physical activity (AOR: 0.32; CI: 0.12–0.84).

### Fairlie decomposition analysis of the contributing factors

Table 4 shows the findings of detailed decomposition of rural–urban differences of depression with multimorbidity among older adults. Percent contribution has been computed by division of each coefficient value of each factor included in the study by the total coefficient value and then multiplied by 100. Results from the decomposition analysis found that education and caste explained 46 percent of the rural–urban inequality in the prevalence of depression with multimorbidity among older adults. ADL disability, IADL disability and self-rated health are the other significant predictors that explained nearly 5 percent, 19 percent and 19 percent of the rural–urban inequality in the prevalence of depression with multimorbidity among older adults respectively. Perceived discrimination and ill-treatment behaviour also significantly explained 24 percent and 5 percent of the rural–urban inequality in the prevalence of depression with multimorbidity among older adults respectively.

**Table 4:** Estimates of rural–urban decomposition analysis for the contribution of explanatory variables of depression among older adults in India, 2017–18

Factors	Coefficient	Standard error	Percent contribution
**Sociodemographic**			
Age	-0.00004	0.00065	-0.11
Sex	0.00135	0.00080	3.36
Education	0.01448	0.00469	35.99**
Religion	-0.00116	0.00041	-2.87**
Caste	0.00414	0.0020	10.30*
**Health and behavioural factors**			
ADL disability	0.00196	0.00046	4.88***
IADL disability	0.00776	0.00160	19.30***
Self-rated health	0.00747	0.00121	18.58***
Pain	0.00040	0.00051	1.00
Body Mass Index	0.00348	0.00277	8.65
Physical activity status	0.00048	0.00104	1.18
Tobacco consumption	0.00093	0.00237	2.31
Alcohol consumption	-0.00314	0.00093	-7.81***
Health insurance coverage	-0.00227	0.00069	-5.64***
**Psycho-social factors**			
Perceived discrimination	0.00976	0.00119	24.25***
Marital Status	-0.00126	0.00049	-3.12*
Working status	-0.00384	0.00195	-9.55*
Ill treatment	0.00208	0.00076	5.18*
Community involvement	0.00025	0.00025	0.62
**Household factors**			
MPCE quintile	-0.00140	0.00105	-3.48
Region	-0.00122	0.00044	-3.03*
**Total**	0.04022		
**Differences (Rural- urban gap)**	0.06468		

^*^p ≤ 0.05; **p ≤ 0.005; ***p ≤ *0.001*; *ADL* Activities of Daily Living, *IADL* Instrumental Activities of Daily Living, *MPCE* Monthly Per Capita Consumption Expenditure

## Discussion

Based on nationally representative data from the LASI wave 1, the current study examined how the place of residence of an individual has influenced the prevalence of depression among older adults with co-morbid conditions in India. The prevalence of depression with multimorbidity among older persons significantly differed by 9.48% between rural and urban areas (38.33% vs 28.85% respectively). Socioeconomic variables such as education, work and caste status, psychosocial variables such as perceived discrimination, health insurance coverage and ill-treatment, and health-related variables such as ADL/IADL disability, self-rated health, experiences of pain and BMI contributed significantly to the rural–urban differences in depression prevalence among older adults with multimorbidity.

By evaluating the prevalence of depression among multimorbid older patients across various socio-demographic and socioeconomic groups, our study made an additional contribution to current knowledge and our findings have potential implications for framing policies. We observed an increased prevalence of depression with multimorbidity especially among participants in higher age groups, females and those with lower levels of education and wealth. The age and gender differentials in the levels of physical discomfort and psychological strain brought on by multimorbidity may explain this predicament, and the possibility of higher financial costs associated with the disease may also lead to greater depression levels among economically poor older people [3, 44]. Clinicians should consider the mental health of older patients especially women and oldest people when managing multimorbidity and provide immediate psychological assistance. Also, it is important to ensure that older adults with multimorbidity have access to an affordable treatment, especially in rural areas, which can significantly enhance their physical and mental well-being [45].

Our findings indicate significant variations in the association between self-rated health, ADL disability and experiences of pain with depression among older multimorbid patients in rural and urban areas. Limited healthcare facilities in rural regions can lead to potential delays in addressing physical and mental health conditions, including depression [46]. In contrast, urban areas benefit from better healthcare resources, allowing for prompt interventions influencing the connection between self-rated health and depression in older patients [32]. Increased community engagement and available social resources further contribute to better perceptions of own health. Limited opportunities in rural settings may affect the ability of older individuals to maintain independence in ADL, contributing to feelings of helplessness and depression. Conversely, greater community resources and engagement options in urban settings may positively impact older patients’ ability to manage ADL, potentially serving as a protective factor against depression in multimorbid patients [47]. Additionally, environmental factors, economic disparities, cultural attitudes, and lifestyle variations further contribute to the complex interplay between experiences of pain and depression in older patients [48, 49]. Understanding these multifaceted factors is essential for tailoring interventions and healthcare strategies to address the unique challenges faced by older patients in rural and urban settings dealing with the complex relationship between pain and depression [50, 51].

Furthermore, we found that older adults with multimorbidity living in both urban and rural areas reported higher levels of depression when they had no insurance coverage, experienced discrimination or ill-treatment, and the rates were higher in rural areas, which is in line with previous studies [52, 53]. This led us to the assumption that older adults who lived in rural areas tend to receive treatment for their chronic illnesses and, therefore, more likely to experience depressive symptoms, which was in line with the study of Keats, M [54]. The gender-based findings of our study imply that the prevalence of depression among multimorbid older women in rural areas may be linked to the role overload, which arises from a combination of work and domestic responsibilities [19]. Similar findings were reported in other cross-sectional studies in the US using data from the National Health Interview Survey (NHIS) [20]. The authors of the study posited that rural residents are more likely to exhibit characteristics associated with depression, such as poverty, chronic diseases, limitations in daily activities, and poor health status [20]. In contrast, a study in Canada revealed that the risk factors associated with depressive symptoms among adults aged 45–85 were somehow similar in rural and urban areas [23]. The findings suggest the need to research further "area-sensitive" healthcare interventions to have deeper understanding of social and environmental factors to the double burden of chronic conditions and mental illnesses in rural areas [55].

Decomposition analysis revealed that gender and caste are the two important factors contributing to the rural–urban difference in the prevalence of depression among multimorbid older patients. Similar to our findings, another study revealed that sex strongly influenced the association between multimorbidity and psychological well-being, and women with multimorbidity were more likely to experience depression [56]. Previous studies also revealed that women had a higher probability of experiencing psychological distress [57, 58]. A number of factors cause the significant adverse effects of multimorbidity on depression in older Indian women. One explanation for gender disparities is that women may be more predisposed to chronic diseases than men when they live in poverty, which increases the risk of further illnesses and the disease overburdening which ultimately lead to elevated depressive symptoms. Traditional Indian culture may also be an important factor that could explain the gender differences in depression among older patients in rural and urban areas. According to India's traditional culture, men should be the ones to participate in community-related meetings, while women should handle most household matters for the family [59]. When older women adequately care for themselves and their families, they have little time left over for socializing, which increases the demand for their time off. As a result, they find it challenging to manage their stress associated with chronic diseases, which leaves them vulnerable to mental health issues. Because of this, women with multimorbidity are more vulnerable than males to experiencing mental health issues.

BMI is also another important contributor to rural–urban differences in the prevalence of depression with multimorbidity among older adults, which is in accordance with the growing body of research [60–64]. This may be due to a range of factors, including limited access to healthcare services [65], social isolation [66], and greater exposure to environmental stressors [67, 68]. Research has shown that obesity is associated with an increased risk of depression, as well as a range of other health conditions such as diabetes, cardiovascular disease, and cancer [69]. However, we found an increased prevalence of depression among underweight older multimorbid patients in rural areas but not in urban areas. Overall, the urban–rural difference in the relationship between obesity and depression among multimorbid older patients is complex and multifactorial. More research is needed to better understand the underlying mechanisms driving these differences and to develop targeted interventions to reduce the burden of depression and multimorbidity in both rural and urban communities.

The strength of the study includes the reasonably large study samples, repeated measures, and validated questionnaires used to assess depression with multimorbidity among older adults. Another advantage is that we also compared the prevalence of depression among people with multiple morbidities in rural and urban settings. This finding may have significant medical implications for preventing and treating depression in older Indian adults. However, some limitations are also there. First, a number of people were excluded from the LASI survey either institutionalized or bedridden and may have multimorbidity and are more vulnerable to depression. Second, the CESD-10 was used to identify depression, which is not considered a clinical diagnosis and could contribute to misclassification bias given different cut points used in different studies for the probable depression. However, epidemiology research among older adults in India has shown that the CESD-10 is reliable and valid, as many studies have used this scale to measure depression [18, 70]. Third, the cross-sectional nature of the study does not allow any causal associations in this study; and there can be bidirectional or reciprocal associations, for example, between self-rated health and depression among multimorbid older patients. Fourth, self-reported data may make depression more likely to be misreported. In India, older people who need financial and physical help in their later years rely on their relatives. As a result, older adults could be afraid to disclose their mental health status in front of their relatives during the investigation. Moreover, due to the lack of data, we were unable to adjust factors like antidepressant medication use. Future research is required to confirm the findings considering these limitations.

## Conclusions

The findings of the study shed light on the prevalence of depression among multimorbid older patients is a significant health concern that affects both rural and urban populations. However, there are notable differences between these two groups that have important implications for healthcare policy and delivery. Overall, older patients living in rural areas tend to experience higher rates of depression than those living in urban areas. This can be attributed to a variety of factors, including higher rates of disability, poor perceived health and limited access to healthcare services due to non-coverage of health insurance and discrimination, and lower social status. Additionally, rural communities often lack the resources and infrastructure necessary to adequately address the mental health needs of older patients.

In contrast, older adults with multimorbidity living in urban areas tend to have greater access to healthcare services and a wider range of support systems, which can help mitigate the risk factors of depressive symptoms. However, urban areas also have their own unique challenges, such as higher rates of economic inequalities and crime, which can exacerbate mental health issues. Given these differences, it is clear that a one-size-fits-all approach to addressing depression among older patients is not effective. Instead, healthcare policies and delivery systems must be tailored to the specific needs of each population. This may involve increasing access to mental health services in rural areas, developing community-based support systems, and addressing social determinants of health such as poverty and social exclusion. In addition, further research is needed to better understand the complex interplay among factors associated with depression in multimorbid persons, and rural–urban differences among those patients. Ultimately, addressing depression with multimorbidity among older adults in rural and urban areas will require a coordinated effort from policymakers, healthcare providers, and community organizations. By working together, we can develop effective solutions that reduce the risk factors of chronic conditions and depression, and improve the quality of life for older adults in all communities.

## Data Availability

The data are publicly available on the website, https://g2aging.org/app/auth/signin?next=/app/lasi/download
